# Unilateral Graves’ Orbitopathy Followed by Hashimoto’s Hypothyroidism: Highlighting Micronutrient Influence in Thyroid Autoimmunity

**DOI:** 10.7759/cureus.90609

**Published:** 2025-08-20

**Authors:** Marjeta Kermaj, Megi Lekbello, Megi Blushi, Dorina Ylli, Agron Ylli

**Affiliations:** 1 Endocrinology Department, University Hospital Center "Mother Tereza", Tirana, ALB; 2 Endocrinology Department, Faculty of Medicine, University of Medicine, Tirana, Tirana, ALB

**Keywords:** graves disease, hashimoto thyroiditis, iron deficiency, thyroid eye disease, vitamin d deficiency

## Abstract

We present the case of a 19-year-old female who initially exhibited unilateral orbital symptoms and laboratory evidence of autoimmune thyroid involvement, consistent with a diagnosis of euthyroid Graves’ orbitopathy (GO). She responded well to immunosuppressive therapy and remained clinically stable for two years. Subsequently, she developed fatigue, menstrual irregularities, and mild recurrent eye discomfort. Laboratory evaluation revealed biochemically severe hypothyroidism with elevated thyroid-stimulating hormone (TSH), low free thyroxine (FT4), and persistently high thyroid peroxidase antibodies (anti-TPO). Additional findings included iron deficiency anemia and vitamin D deficiency. Thyroid ultrasound confirmed features of autoimmune thyroiditis, and she was started on hormonal and micronutrient replacement therapy. This case highlights a rare clinical evolution from euthyroid GO to Hashimoto’s thyroiditis (HT) and emphasizes the need for long-term follow-up in autoimmune thyroid conditions. It also suggests that micronutrient status may influence immune shifts and disease progression, underscoring the importance of addressing modifiable factors as part of comprehensive endocrine care.

## Introduction

Hashimoto’s thyroiditis (HT) and Graves’ disease (GD) are the two most common autoimmune disorders affecting the thyroid gland. GD typically leads to an overactive thyroid (hyperthyroidism), while HT results in an underactive thyroid (hypothyroidism) [1]. Although traditionally considered distinct entities, they share overlapping immune mechanisms, including autoreactive T-cell activity and thyroid-specific autoantibodies [2].

In rare cases, a clinical transition from GD to HT can occur, reflecting the dynamic and evolving nature of autoimmune thyroid disease (AITD) [3]. Such transitions pose diagnostic and therapeutic challenges, particularly when accompanied by thyroid-associated orbitopathy (TAO), an inflammatory condition affecting orbital tissues.

We present the case of a 19-year-old female who developed biochemically severe hypothyroidism two years after a confirmed diagnosis of unilateral TAO related to GD. In addition to immune dysregulation, emerging evidence suggests that micronutrient deficiencies, particularly in iron and vitamin D, may influence the trajectory and activity of AITD. This case underscores the importance of a comprehensive and individualized approach to AITD, taking into account not only immunological fluctuations but also modifiable metabolic factors, even in young patients.

## Case presentation

**Figure 1:**
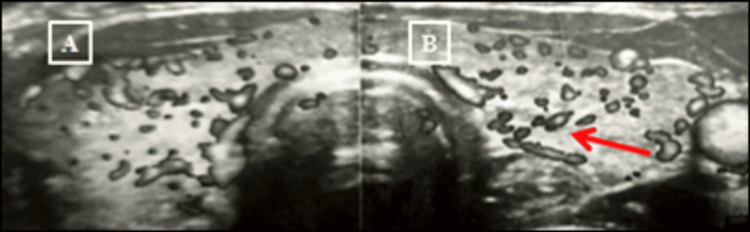
Thyroid ultrasound with features of autoimmune thyroiditis (A) Transverse ultrasound image of the right thyroid lobe; (B) Transverse ultrasound image of the left thyroid lobe. Both lobes exhibit a hypoechoic, inhomogeneous echotexture with increased vascularity, consistent with autoimmune thyroiditis. The red arrow highlights the vascular pattern.

**Figure 2:**
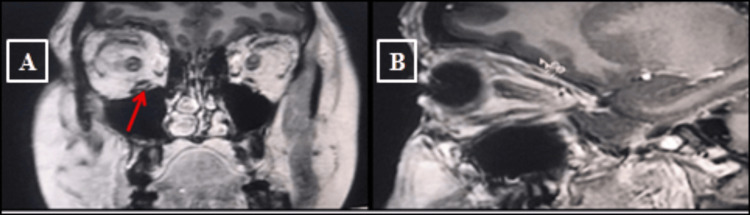
Coronal and sagittal CT scans of the orbits showing enlargement of the right inferior rectus muscle without optic nerve compression . (A) Coronal CT scan of the orbits showing enlargement of the right inferior rectus muscle (highlighted by the red arrow); (B) Sagittal CT view confirming focal hypertrophy of the same muscle. The optic nerves and globes appear normal. These findings are consistent with unilateral thyroid-associated orbitopathy (TAO) and underlying Graves’ disease.

A 19-year-old female presented to our department with progressive fatigue, cold intolerance, and right-sided eye swelling. The patient reported no family history of thyroid or autoimmune disease, no recent psychological stress, and no history of smoking. She also denied use of iodine-containing supplements or medications. Notably, two years earlier, she had consulted an endocrinologist due to persistent ocular discomfort on the right side. Clinical examination at that time raised suspicion of unilateral thyroid-associated orbitopathy (TAO), related to GD. As part of the initial workup two years prior, investigations were conducted, including serum thyroid-stimulating hormone (TSH), anti-TSH receptor antibodies (anti-TSH-R), thyroid peroxidase antibodies (anti-TPO), thyroid ultrasound, and a head CT scan. At that time, thyroid ultrasound revealed a heterogeneous and hypoechoic gland with signs of mild hypervascularization, consistent with autoimmune thyroiditis (Figure 1). A coronal and sagittal CT scan of the orbits showed enlargement of the right inferior rectus muscle, with no optic nerve compression (Figure 2). Laboratory findings from that period are summarized in Table 1.

**Table 1:** The results of the laboratory tests TSH: thyroid-stimulating hormone, TSH-R: TSH receptor, FT4: free T4, FT3: free T3, TPO: thyroid peroxidase

Parameter (unit)	Result	Normal range
TSH (mIU/L)	2.76	0.7-6.4
anti TSH-R (U/I)	2.12	<1.0
FT4 (ng/dl)	0.86	0.7-1.48
FT3 (pg/ml)	3.2	2.31-3.71
anti-TPO (IU/ml)	95.96	<30

The results showed a normal TSH level (2.76 mIU/L), which may appear atypical in a patient with suspected thyroid eye disease (TED). However, anti-TSH-R were mildly elevated at 2.12 U/I (normal <1.0), which is suggestive of autoimmune stimulation and supports the diagnosis of early or evolving GD. Free T4 (FT4) and free T3 (fT3) levels were within normal limits, consistent with an euthyroid state at the time of testing. Anti-TPO antibodies were significantly elevated (95.96 IU/mL, normal <30), confirming the presence of autoimmune thyroiditis.

An ophthalmologic assessment revealed intermittent diplopia, mild eye dryness, and increased ocular tension in the right eye. Conjunctival redness and upper eyelid swelling were present. Exophthalmos was measured using a Hertel exophthalmometer, with readings of 20 mm in the right eye and 16 mm in the left, corresponding to an intercanthal distance of 104 mm. Visual acuity was 10/10 bilaterally, and both color vision and fundus examination were normal. Retraction of the lower lateral eyelid on the right side was noted. The Clinical Activity Score (CAS) was 3 [4], which, according to European Group on Graves’ Orbitopathy (EUGOGO) criteria (CAS ≥3/7), indicates active disease. Due to the presence of intermittent diplopia, ocular hypertension, and anatomical changes (proptosis and lid retraction), the disease was classified as moderate-to-severe in severity, warranting immunosuppressive treatment according to the 2021 EUGOGO protocol [5].

She was admitted and treated with intravenous methylprednisolone acetate, receiving a total cumulative dose of 4.5 g over 12 weeks. Ocular symptoms improved significantly after treatment.

Two years later, the patient re-presented with symptoms of photophobia and tearing, but no signs of active inflammation were observed. CAS was 0, indicating inactive orbitopathy.

Laboratory tests revealed mild iron deficiency anemia (Table 2) and biochemically severe hypothyroidism. TSH was markedly elevated at 95.32 mIU/L (normal range: 0.35-4.94), and FT4 was significantly decreased at 0.55 ng/dL, confirming overt hypothyroidism. FT3 was also reduced (2.10 pg/mL), consistent with central metabolic slowdown. Anti-TPO antibodies were markedly elevated (889.10 IU/mL), highly suggestive of HT. Ferritin levels were low (15 ng/mL), which explains the iron deficiency anemia, while 25-hydroxy-vitamin D levels were also low (12 ng/mL), indicating a vitamin D deficiency (Table 3).

**Table 2:** Complete blood count revealing mild iron deficiency anemia

Parameter (Unit)	Result	Normal range
RBC (×10⁶/µL)	4.54	4.4 - 5.9
Hemoglobin (Hgb) (g/dL)	11.3	13 - 17
Hematocrit (HCT)(%)	36.0	42 - 52
Mean Corpuscular Volume (MCV) (fL)	79.3	80 - 100
Mean Corpuscular Hemoglobin (MCH) (pg)	25.0	27 - 34
Mean Corpuscular Hemoglobin Concentration (MCHC) (g/dL)	31.5	32 – 36
Red Cell Distribution Width (RDW) (%)	15.6	10.8 - 14
White Blood Cells (WBC) (K/µL)	6.4	4 - 10.5
Neutrophils (K/µL)	3.1	1.6 - 7.56
Lymphocytes (K/µL)	1.8	1 - 4.72
Platelets (PLT) (K/µL)	372	150 – 400

**Table 3:** Hormonal, immunologic and micronutrient profile at follow-up evaluation TSH: thyroid-stimulating hormone, TPO: thyroid peroxidase

Parameter (Unit)	Result	Normal range
TSH (mIU/L)	95.32	0.35-4.94
Free T4 (FT4) (ng/dL)	0.55	0.7-1.48
Free T3 (FT3) (pg/mL)	2.10	2.31-3.71
Anti-TPO (IU/mL)	889.10	<5.5
Cortisol (µg/dL)	21.4	3.7-19.4
Follicle-Stimulating Hormone (FSH) (IU/L)	6.16	0.3-7.8
Luteinizing Hormone (LH) (IU/L)	13.62	0.1-8.4
Ferritin (ng/mL)	15	13-150
Vitamin D3 (ng/mL)	12	<20 insufficiency

**Figure 3:**
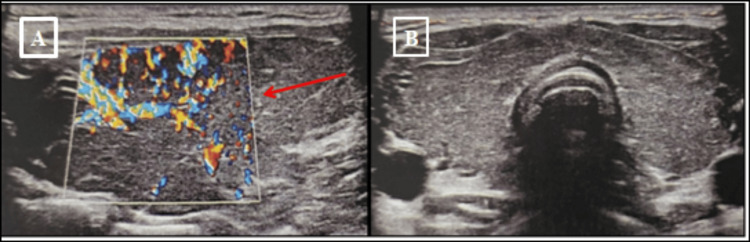
Thyroid ultrasound showing a hypoechoic and inhomogeneous gland with increased vascularity, as demonstrated by color Doppler imaging on the left (highlighted by the red arrow), and a transverse view on the right confirming features of autoimmune thyroiditis. (A) Longitudinal Doppler ultrasound of the thyroid showing increased intraparenchymal vascularity (highlighted by the red arrow), a typical feature of autoimmune thyroiditis; (B) Transverse grayscale ultrasound image of the thyroid gland confirming diffuse hypoechoic and heterogeneous echotexture, consistent with autoimmune involvement.

Thyroid ultrasound revealed a hypoechoic, heterogeneous gland with diffuse vascularity, consistent with features of autoimmune thyroiditis (Figure 3). 

The final diagnoses included inactive, moderate unilateral TAO in the right eye, biochemically severe hypothyroidism due to HT, iron deficiency anemia, and vitamin D deficiency. The patient was started on levothyroxine (75 mcg/day), oral ferrous sulfate, vitamin D3 (5000 IU/day), and artificial tears.

At follow-up, thyroid function normalized, and ocular symptoms remained stable. Laboratory monitoring revealed a gradual correction of iron and vitamin D levels, which paralleled clinical improvement in fatigue and overall well-being. The patient tolerated the treatment well, with no adverse effects reported.

## Discussion

TAO is a well-recognized manifestation of GD, typically linked to hyperthyroidism and the presence of thyroid-stimulating antibodies (TSAb) [6]. However, atypical presentations such as unilateral orbitopathy or TAO in hypothyroid or euthyroid individuals have been reported, albeit rarely, and are occasionally seen in association with HT [7].

Our case highlights an unusual clinical evolution, from unilateral TAO associated with GD to overt hypothyroidism two years later, in the absence of ablative therapy, raising the possibility of an underlying immune phenotype shift within the spectrum of AITD. Transitions between GD and HT have been described in the literature and are thought to result from changing patterns in immune dominance, shifting from stimulating TSAb to blocking or destructive TSH receptor antibodies (TSBAb) [8].

In our patient, the presence of mildly elevated anti-TSH-R antibodies (2.12 U/I) during the initial phase was suggestive of early Graves' activity, while the subsequent marked elevation of anti-TPO antibodies (889 IU/mL) was confirmatory for HT. This evolving antibody profile supports a shift from immune stimulation to glandular destruction, a hallmark of the GD-to-HT transition.

Although both anti-TSH-R and anti-TPO antibodies are markers of AITD, they reflect distinct pathogenic pathways. Anti-TSH-R antibodies are commonly associated with orbital inflammation and hyperthyroidism, while anti-TPO antibodies are linked to thyrocyte destruction and hypothyroidism. The coexistence of both antibody types in our case underscores the dual autoimmune mechanism potentially driving both ocular and thyroidal involvement.

Genetic and immunologic overlap between GD and HT is well-established, with shared susceptibility loci such as HLA-DR3, CTLA-4, and PTPN22. However, disease-specific loci like VAV3 have also been implicated in HT [9,10]. Histopathological and pediatric studies have demonstrated lymphoid infiltration and follicular destruction during early phases of thyrotoxicosis, which may mimic or evolve into HT-like features [11,12]. Immunophenotyping of thyroid tissue in GD and HT reveals a common immune architecture, including tertiary lymphoid structures and germinal centers, supporting the notion of a spectrum model of AITD.

Brancatella et al. also demonstrated how antibody profiles and ultrasonographic features correlate with distinct histopathology in GD, highlighting the phenomenon of immune remodeling over time [13]. In our case, the initial presentation with unilateral orbitopathy and mild anti-TSH-R antibody elevation was highly suggestive of early GD. Although TAO is more commonly bilateral and hyperthyroid-associated, its unilateral form, particularly in young individuals, requires careful diagnostic evaluation.

The relationship between anti-TSH-R antibody titers and TAO severity has been extensively studied, with high antibody levels correlating strongly with the development and persistence of orbitopathy [14]. In our patient, the initial CAS was 3, classifying the disease as clinically active but moderate-to-severe based on functional and anatomical findings. At re-presentation two years later, the CAS was 0, confirming clinical remission of orbitopathy and underscoring the value of long-term follow-up.

A noteworthy aspect of this case is the presence of iron and vitamin D deficiencies, both of which have known immunomodulatory roles. Iron is a critical cofactor for the enzyme TPO, and its deficiency may impair thyroid hormone synthesis and promote autoimmune reactivity. In our patient, low ferritin (15 ng/mL) and mild anemia may have contributed to persistent fatigue and elevated anti-TPO antibody levels [15].

Similarly, vitamin D deficiency (12 ng/mL) likely played a role in immune dysregulation, as vitamin D is essential for maintaining self-tolerance via regulatory T cell modulation. Numerous studies have linked hypovitaminosis D to increased risk and severity of AITD [16]. Selenium, although not administered in this case, has been shown to reduce anti-TPO antibody titers and improve outcomes in HT. At our institution, selenium supplementation is not routinely recommended unless deficiency or active orbitopathy is confirmed [17].

Although the patient did not report gastrointestinal symptoms, the possibility of subclinical autoimmune enteropathy or malabsorption contributing to micronutrient deficiency cannot be excluded. In similar cases, a broader immunological workup, including screening for celiac disease or autoimmune polyglandular syndromes, may be warranted [18].

In summary, this case illustrates the dynamic nature of AITD, including the rare transition from GO to HT-related hypothyroidism, without definitive therapy. It emphasizes the need for long-term immunologic surveillance, careful interpretation of antibody profiles, and recognition of modulating factors such as iron and vitamin D status in shaping autoimmune trajectories.

## Conclusions

This case illustrates a rare clinical transition from GD to HT in the context of persistent orbitopathy. It underscores the importance of long-term monitoring of thyroid function and autoimmunity, even after apparent clinical stabilization. Moreover, it highlights the potential influence of modifiable factors, such as iron and vitamin D deficiencies, on the course of AITD. Given their plausible role in disease progression, regular screening and correction of micronutrient deficiencies should be integrated into endocrine care as a simple yet impactful strategy to support immune balance and reduce the risk of relapse. A patient-centered, integrative follow-up approach remains essential for managing complex and dynamic autoimmune presentations.

## References

[1] Antonelli A, Ferrari SM, Corrado A, Di Domenicantonio A, Fallahi P (2015). Autoimmune thyroid disorders. Autoimmun Rev.

[2] Weetman AP (2000). Graves' disease. N Engl J Med.

[3] McLachlan SM, Nagayama Y, Pichurin PN (2007). The link between Graves' disease and Hashimoto's thyroiditis: a role for regulatory T cells. Endocrinology.

[4] Mourits MP, Koornneef L, Wiersinga WM, Prummel MF, Berghout A, van der Gaag R (1989). Clinical criteria for the assessment of disease activity in Graves' ophthalmopathy: a novel approach. Br J Ophthalmol.

[5] Bartalena L, Kahaly GJ, Baldeschi L (2021). The 2021 European Group on Graves' orbitopathy (EUGOGO) clinical practice guidelines for the medical management of Graves' orbitopathy. Eur J Endocrinol.

[6] Bahn RS (2010). Graves' ophthalmopathy. N Engl J Med.

[7] Ponto KA, Binder H, Diana T (2015). Prevalence, phenotype, and psychosocial well-being ineuthyroid/hypothyroid thyroid-associated orbitopathy. Thyroid.

[8] McLachlan SM, Rapoport B (2013). Thyrotropin-blocking autoantibodies and thyroid-stimulating autoantibodies: potential mechanisms involved in the pendulum swinging from hypothyroidism to hyperthyroidism or vice versa. Thyroid.

[9] Tomer Y, Davies TF (2003). Searching for the autoimmune thyroid disease susceptibility genes: from gene mapping to gene function. Endocr Rev.

[10] Oryoji D, Ueda S, Yamamoto K (2015). Identification of a Hashimoto thyroiditis susceptibility locus via a genome-wide comparison with Graves' disease. J Clin Endocrinol Metab.

[11] Sato T, Takata I, Taketani T, Saida K, Nakajima H (1977). Concurrence of Grave's disease and Hashimoto's thyroiditis. Arch Dis Child.

[12] Buckingham BA, Costin G, Kogut MD (1977). Pathologic and immune factors in thyroid disease. J Pediatr.

[13] Brancatella A, Torregrossa L, Viola N (2023). In Graves' disease, thyroid autoantibodies and ultrasound features correlate with distinctive histological features. J Endocrinol Invest.

[14] Kahaly GJ, Wüster C, Olivo PD, Diana T (2019). High titers of thyrotropin receptor antibodies are associated with orbitopathy in patients with Graves' disease. J Clin Endocrinol Metab.

[15] Garofalo V, Condorelli RA, Cannarella R, Aversa A, Calogero AE, La Vignera S (2023). Relationship between iron deficiency and thyroid function: asystematic review and meta-analysis. Nutrients.

[16] Czarnywojtek A, Florek E, Pietrończyk K (2023). The role of vitamin D in autoimmune thyroid diseases: a narrative review. J Clin Med.

[17] Gärtner R, Gasnier BC, Dietrich JW, Krebs B, Angstwurm MW (2002). Selenium supplementation in patients with autoimmune thyroiditis decreases thyroid peroxidase antibodies concentrations. J Clin Endocrinol Metab.

[18] Kahaly GJ (2009). Polyglandular autoimmune syndromes. Eur J Endocrinol.

